# The effect of pharmacological treatment on gait biomechanics in peripheral arterial disease patients

**DOI:** 10.1186/1743-0003-7-25

**Published:** 2010-06-07

**Authors:** Jessie M Huisinga, Iraklis I Pipinos, Jason M Johanning, Nicholas Stergiou

**Affiliations:** 1 Nebraska Biomechanics Core Facility, University of Nebraska at Omaha, 6001 Dodge Street Omaha, NE, USA 68182, USA; 2 Department of Surgery, University of Nebraska Medical Center, 983280 Nebraska Medical Center, Omaha, NE, USA 68198-3280, USA; 3 Department of Environmental, Agricultural, and Occupational Health, College of Public Health, University of Nebraska Medical Center, 986075 Nebraska Medical Center, Omaha, NE, USA 68198-6075, USA

## Abstract

**Background:**

Pharmacological treatment has been advocated as a first line therapy for Peripheral Arterial Disease (PAD) patients suffering from intermittent claudication. Previous studies document the ability of pharmacological treatment to increase walking distances. However, the effect of pharmacological treatment on gait biomechanics in PAD patients has not been objectively evaluated as is common with other gait abnormalities.

**Methods:**

Sixteen patients were prescribed an FDA approved drug (Pentoxifylline or Cilostazol) for the treatment of symptomatic PAD. Patients underwent baseline gait testing prior to medication use which consisted of acquisition of ground reaction forces and kinematics while walking in a pain free state. After three months of treatment, patients underwent repeat gait testing.

**Results:**

Patients with symptomatic PAD had significant gait abnormalities at baseline during pain free walking as compared to healthy controls. However, pharmacological treatment did not produce any identifiable alterations on the biomechanics of gait of the PAD patients as revealed by the statistical comparisons performed between pre and post-treatment and between post-treatment and the healthy controls.

**Conclusions:**

Pharmacological treatment did not result in statistically significant improvements in the gait biomechanics of patients with symptomatic PAD. Future studies will need to further explore different cohorts of patients that have shown to improve significantly their claudication distances and/or their muscle fiber morphology with the use of pharmacological treatment and determine if this is associated with an improvement in gait biomechanics. Using these methods we may distinguish the patients who benefit from pharmacotherapy and those who do not.

## Introduction

Peripheral Arterial Disease (PAD) is a condition of atherosclerosis affecting the arteries of the lower extremities which results in reduced blood flow at rest with further reduction occurring with activity. Currently, PAD affects 12% of the general U.S. population and up to 20% of individuals 75 years and older [1]. During walking, PAD patients typically experience intermittent claudication symptoms. These symptoms include muscle aching and cramping secondary to ischemia in the calf, thigh, or buttocks [1]. It has been shown that intermittent claudication is associated with alterations of basic temporal-spatial (i.e. speed, step length) gait characteristics [2-4], changes in functional status [5,6], increased risk of other poor health outcomes [7], and increased risk for falls [7-10]. The underlying cause of gait abnormalities in patients with symptomatic PAD is not simply blood flow restriction. Also associated is the chronic ischemia reperfusion cycle which results in mitochondriopathy and reactive oxygen species damage leading to neuromuscular damage of the lower extremity [11,12]. Importantly, these histological changes in the mitochondria are then present in the absence of ischemia.

Despite our knowledge regarding the patho-physiology of symptomatic PAD, pharmacological therapies are limited. Currently, only two medications are approved by the FDA for treatment of intermittent claudication secondary to PAD. The older of the two, pentoxifylline, acts by altering the hemorheological properties of blood leading to reduced blood viscosity and hypercoagulability [13]. Research has found that pentoxifylline can improve the respiration capacity of the mitochondria which may result in changes in muscle physiology during physical activity [14]. The other, cilostazol, increases the intracellular concentration of the cyclic adenosine monophosphate in order to suppress platelet aggregation and increase arterial dilation [15]. Although the purpose of these medications is to eliminate symptoms and improve the distance walked by patients with symptomatic PAD, changes in the biomechanics of gait have not been documented as a result of pharmacological treatment in PAD patients [16]. Especially, it is unknown if such treatment can improve the biomechanics of gait of PAD patients towards the level of normative healthy gait.

Recently, studies have been performed where biomechanical measures have been used to identify differences between PAD patients and healthy controls [2,3,17-20]. Initial evaluations have clearly identified specific gait deficiencies in PAD patients emphasizing the importance of biomechanical measures to fully characterize the gait handicap in these patients and delineate the underlying mechanisms of this disease. In the present study, we extended previous work utilizing the same kinematic and kinetic biomechanical measures to assess the impact of pharmacological treatment of PAD patients.

Therefore, the purpose of this study was to determine the impact of pharmacological treatment on the biomechanics of gait of PAD patients as compared to healthy controls. Biomechanical parameters have been successfully utilized to identify the effect of pharmacological therapies in other gait related disorders such as osteoarthritis [21]. Thus, examining the effect of pharmacological treatment is an important step in evaluating the effectiveness of patient treatment. We hypothesized that pharmacological treatment would produce changes in the kinematics and kinetics of gait in PAD patients. It was also hypothesized that differences would be present in PAD patients as compared to healthy controls in the pre-treatment collection and these differences would be reduced in the post-treatment collection. By determining whether gait function improves as a result of pharmacological treatment, it will be possible to design a more effective treatment plan.

## Methods

### Subject inclusion and exclusion criteria

A total of 16 PAD patients and 14 healthy control subjects matched in age, mass, height, BMI, and gender volunteered to participate in this study (Table 1). The participation of 16 PAD patients resulted in 30 total limbs included for analysis, with two patients having unilateral symptoms. From the 14 healthy controls, all 28 limbs were used. The PAD patients received either pentoxifylline or cilostazol as treatment for intermittent claudication. Cilostazol and pentoxifylline are both approved by the FDA for treatment of claudication pain. PAD patients were assigned to one of the pharmacological agents at the discretion of the treating physician based on the patient's medical history and participating medication formulary. Therefore, the drug assignment within the study was not random and the investigators were not blinded to the treatment. All patients were treatment naïve with regards to both cilostazol and pentoxifylline. All patients were evaluated and treated in a standard fashion for non-invasive treatment of symptomatic PAD. The study was not specifically designed to compare pentoxifylline to cilostazol, but instead to examine the overall effect of pharmacotherapy on the biomechanics of gait of PAD patients as compared to healthy controls. Therefore, all patients were grouped together for analysis without regard for the pharmacological agent being taken.

**Table 1:** Baseline characteristics of PAD patients and healthy control subjects.

	Patient (N = 30 limbs)	Control (N = 28 limbs)
**Clinical Characteristics**		

Gender (Male/Female)	15/1	13/1
Age (years)	65.8 ± 9.51	64.7 ± 10.3
Body mass (kg)	79.97 ± 14.90	81.16 ± 21.45
Body height (m)	1.71 ± .46	1.73 ± .88
BMI	27.28 ± 4.99	26.9 ± 5.34
ABI		
Right	0.55 ± 0.14	>.90
Left	0.60 ± 0.20	>.90
Hypertension (%)	75	0
Smoking (%)	31.3	0
Hyperlipidemia (%)	75	0
Diabetes mellitus (%)	0	0

Note: ABI is the ratio of systolic pressure at the posterior tibial and dorsal pedis artery in the ankle and the brachial artery in the arm where an ABI < 0.9 is an indication of PAD.

PAD patients were recruited at the Nebraska Department of Veterans Affairs (VA) hospital by two board certified vascular surgeons (coauthors I.P., J.J.). Patients were specifically evaluated prior to study enrollment to ensure that walking impairments were secondary to claudication pain. Patients with ambulation limiting cardiac, pulmonary, neuromuscular, or musculoskeletal disease or those who experienced pain or discomfort during walking for reasons other than claudication, such as arthritis, low back pain, or other orthopedic problems, were excluded. Additional exclusion criteria for the PAD patients were severe congestive heart failure, severe hypertension (>180/110), severe lung disease, severe ischemic heart disease, severe arthritis, threatened limb loss (foot ulcers or gangrene), uncontrolled hyperlipidemia or any other process limiting the ability to walk.

The control subjects were recruited from the community and were screened to exclude vascular disease. Ankle brachial index (ABI), which is the ratio of systolic pressure at the posterior tibial and dorsal pedis artery in the ankle and the brachial artery in the arm, was measured to confirm the level greater than 0.9 as part of inclusion criteria where ABI < 0.9 indicates a PAD diagnosis. Control subjects were screened in a similar manner as the PAD patients and were excluded for the same ambulation limiting problems. All participating patients gave informed consent in the presence of one of the vascular surgeons when they were seen and evaluated in the vascular clinic, while control subjects gave informed consent in the presence of one of the secondary investigators at the time of the data collection appointment. All procedures were approved by the University of Nebraska Medical Center and the local VA institutional review boards.

### Experimental procedure and data collection

For all data collections, subjects arrived at the Biomechanics Laboratory and were prepared for data collection by wearing a form fitting outfit and obtaining height, body weight, and anthropometric data. Reflective markers were placed bilaterally according to anatomical position and a modified Helen Hayes marker set [22]. Patients walked through the 10 meter walk-way at a normal pace without care of the position of the force platform. Then they were asked to sit and rest for one minute before and after each walking trial. The rest period was mandatory to insure all trials were without ischemia and that patients did not experience any claudication pain. We collected only one limb at a time since only one force platform is available in the laboratory, thus justifying the usage of both limbs for our data analysis. The limb collected first was randomly selected to insure fatigue was not a factor in the results. Data were collected from heel contact to toe off on the force platform, representing an entire stance cycle. Five trials were collected from each leg for a total of ten trials. On average patients completed a total of 15 walkovers in order to obtain the ten successful trials.

Absolute claudication distance was measured at the end of the data collection after a period of five minutes of rest to insure the beginning of test commenced while the patients were pain-free. Patients walked on a treadmill at a speed of 0.67 m/s and at a grade of 10% according to published clinical guidelines [23]. Patients walked until they were unable to continue due to claudication pain, while the times of onset of claudication pain and absolute claudication pain, which caused stoppage of ambulation, were recorded.

The data collection procedure for the PAD patients was identical for the pre-and post-pharmacological treatment visit. The two collection times were separated by three months to ensure medication effect to be fully present and because three months is the same treatment period used in previous pharmacological studies with PAD patients [24,25]. Data collection procedure for the control patients was identical to the procedure used for the PAD patients. Control subjects, however, only had one data collection performed with no repeat test. Ground reaction forces were acquired using a floor mounted Kistler force plate (Kistler 9281 B, Kistler Instrumentation Corporation, Amhurst, NY) sampling at 600 Hz. The positions of the reflective markers were captured using a six camera system (6 camera Eagle system, Motion Analysis Corp., Santa Rosa, CA) sampling at 60 Hz. From the ground reaction force data and the positions of the markers, joint kinetics and kinematics were calculated from the sagittal plane of motion during the stance phase of walking. A low-pass fourth-order Butterworth filter with a 6 Hz cutoff was used to smooth the marker trajectories during post data processing. Relative joint angles were calculated by the methods described by Vaughn et al. [26] and Nigg et al. [27]. A custom MatLab program was used to calculate the joint kinetics and kinematics of each subject while inverse dynamics using linear and angular Newtonian equations of motion were used to calculate the joint moments at each joint throughout the stance phase of the gait cycle. The joint kinetics parameters were scaled to body weight and body height [28]. The specific dependent variables that were analyzed are listed in Tables 2 and 3. These variables were selected based on previous literature involving the biomechanics of gait of PAD patients and the elderly [2,17,19,29-34].

**Table 2:** Group means and standard deviations for Joint Angle Ranges of Motion; Sig†, p < 0.005.

	PAD pre		PAD post		Control		p-values (effect size)		
	mean	S.D.	mean	S.D.	mean	S.D.	pre/post	pre/con	post/con
AROM	19.96	2.95	19.64	3.07	16.06	3.31	0.669 (0.11)	**< 0.001† **(1.25)	**< 0.001† **(1.12)
KROM	9.18	3.90	9.41	3.58	10.81	3.56	0.839 (0.06)	0.105 (0.43)	0.140 (0.29)
HROM	35.22	4.21	35.98	3.04	39.56	2.87	0.404 (0.21)	**< 0.001† **(1.22)	**< 0.001† **(1.21)

All mean values are in degrees of motion. AROM - Ankle range of motion; KROM - Knee range of motion; HROM - Hip range of motion.

**Table 3:** Group means and standard deviations for Joint Moments; Sig†, p < 0.005.

	PAD pre		PAD post		Control		p-values (effect size)		
(%BWxBH)	mean	S.D.	mean	S.D.	mean	S.D.	pre/post	pre/con	post/con
ADMM	-3.41	0.97	-3.05	0.82	-3.74	1.11	0.030 (0.40)	0.241 (0.32)	0.011 (0.72)
APMM	14.84	1.62	15.54	1.69	15.05	1.57	0.036 (0.42)	0.632 (0.13)	0.266 (0.30)
KFMM	2.00	1.64	1.97	2.04	1.73	1.34	0.912 (0.02)	0.494 (0.19)	0.602 (0.14)
KEMM	-6.59	2.50	-6.61	2.30	-8.42	2.48	0.950 (0.01)	0.009 (0.74)	0.007 (0.76)
HFMM	8.67	3.23	8.21	3.10	11.09	2.40	0.371 (0.14)	**0.003† **(0.86)	**< 0.001† **(1.04)
HEMM	-8.27	2.31	-8.69	2.45	-10.28	2.70	0.160 (0.17)	0.005 (0.80)	0.027 (0.62)

ADMM - Peak ankle dorsiflexion moment during early stance; APMM - Peak ankle plantarflexion moment during late stance; KFMM - Peak knee flexion moment during stance; KEMM - Peak knee extension moment during stance; HFMM - Peak hip flexion moment during late stance; HEMM - Peak hip extension moment during early stance.

All five overground walkovers were used to produce the average for each leg corresponding to each measured variable which was then used for statistical analysis. Independent t-tests were performed to compare the dependent variables of the PAD patients from both pre-treatment and post-treatment to the healthy controls. Dependent student t-tests were used to compare the PAD patients pre-treatment to the post-treatment gait parameters. Statistical analysis was performed using SPSS 12.0. Due to the large number of comparisons (joint angle and moment variables), a Bonferroni correction was employed and the α-level was adjusted to be at 0.005 (0.05/10). Effect size was calculated using the Cohen's d method. Parametric statistics were utilized because gait analysis data have showed to have good normality [35]. Additionally gait data from PAD patients has been shown to exhibit strong tendency for normality among subjects [16].

## Results

### Time-Distance Parameters

Walking speed was significantly less in the PAD patients (p < 0.05) as compared to healthy controls (1.37 ± 0.15) both pre- (1.17 ± 0.11) and post-treatment (1.18 ± 0.16). An average improvement in absolute claudication distance of 20.14 ± 158.7 meters [pre-treatment (236.55 meters) and post-treatment (256.69 meters)] was noted for the group as a whole with pharmacological treatment. However due to the large standard deviations (78.13 meters for pre-treatment and 162.19 meters for post-treatment), there was no significant difference (*p *= 0.619) between pre- and post-treatment for the absolute claudication distance.

### Joint Angles

**Figure 1:**
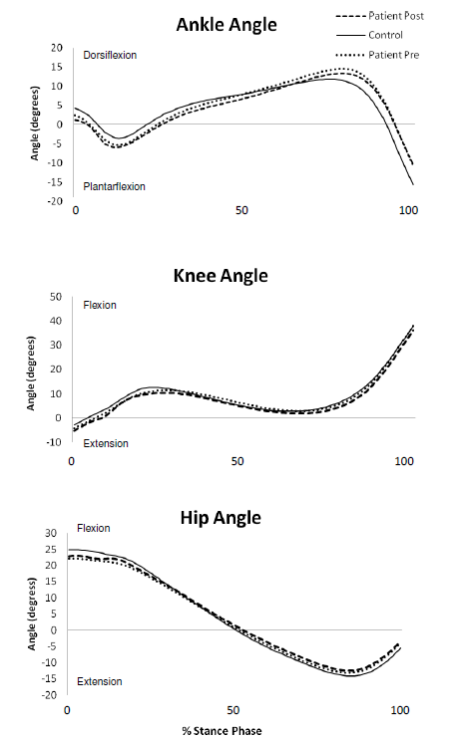
**Mean ensemble curves for Joint Angles**.

Before the application of pharmacological treatment the PAD patients had significantly increased range of motion at the ankle (AROM) (Figure 1; Table 2) and significantly decreased hip range of motion (HROM) as compared to the controls (Figure 1; Table 2). There was no difference in the knee parameters evaluated between the two groups.

After pharmacological treatment, the same significant differences were found between the controls and PAD patients (Table 2). No significant differences were found in the PAD patients comparing pre- and post-pharmacological treatment (Table 2).

### Joint Moments

**Figure 2:**
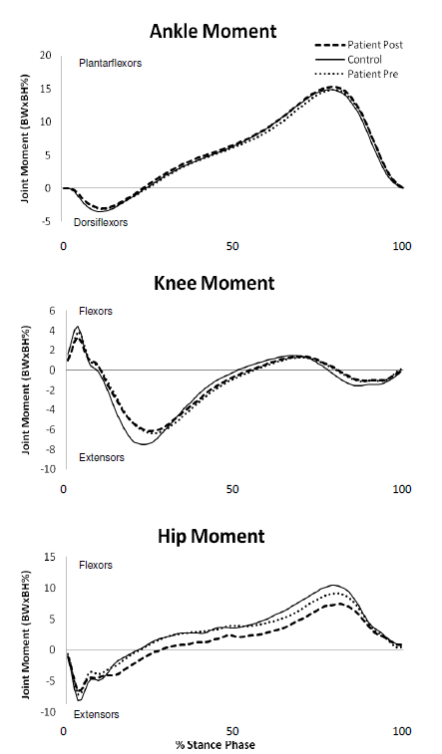
**Mean ensemble curves for Joint Moments**.

Before the application of pharmacological treatment, peak hip flexor moment (HFMM) was significantly decreased in PAD patients compared to healthy controls (Figure 2; Table 3). The adopted α-value being 0.005 after the Bonferroni correction prohibits the identification of some significant differences at the knee and hip. Specifically, the p-values for knee extension moment (KEMM; *p *= 0.009), and hip extension moment (HEMM; *p *= 0.005) are below *p *= 0.01 (Table 3). However, these results are not statistically significant due to the Bonferroni correction used. These outcomes are observable in the graphical representation (Figure 2).

After pharmacological treatment, peak hip flexor moment was still significantly decreased in PAD patients compared to healthy controls (Figure 2; Table 3). Importantly, no significant differences were found in the PAD patients between pre- and post- pharmacological treatment (Table 3).

## Discussion

The purpose of this study was to determine the impact of pharmacological treatment on the biomechanics of gait of PAD patients as compared to healthy controls. By determining whether or not specific biomechanical changes exist as a result of this treatment, it will be possible to determine if pharmacological treatment of patients with PAD exerts an improvement in biomechanical parameters. The pharmacological agents in the study (pentoxifylline and cilostazol) are the two currently approved FDA agents utilized to treat claudication. The medications were considered together because the goal of this study was not to compare the effects of a specific pharmacological treatment but rather to determine if pharmacological treatment resulted in any appreciable change in biomechanical parameters. Comparisons between the PAD patients pre- and post-treatment were conducted with healthy controls. It was anticipated that PAD patients would exhibit a large number of differences as compared to healthy controls in the joint angles and joint moments during walking in the pre-pharmacological treatment phase. It was also anticipated that after the conclusion of the treatment, significant improvements in biomechanical parameters would be seen and the differences between the PAD patients and the healthy controls would decrease and potentially disappear due to the positive effect of the pharmacological treatment.

Our results supported our previous findings that PAD patients have significant gait abnormalities in the absence of claudication pain as compared to control patients even during the absence of ischemia [2,3,19,20,29,30]. However, significant improvements were not found in the gait biomechanics of PAD patients due to pharmacotherapy. Thus, our current evaluation showed that pharmacological treatment did not affect the biomechanics of gait of PAD patients.

Despite a lack of significant differences between the pre- and post-pharmacological treatment conditions, a closer look at the data can provide insight regarding the movement patterns of the PAD patients. When evaluating patients with PAD as compared to control subjects, little data exists to fully quantify the exact biomechanical abnormalities that are present during walking. The evaluation of the joint angles in our study showed that PAD patients exhibited decreased hip flexion as the leg comes in contact with the ground resulting in significantly decreased hip range of motion during stance. The decreased hip flexion indicates that PAD patients position the leg closer to the body as they come in contact with the ground. This is probably an adaptive strategy employed by the PAD patients because the closer the leg is to the body, the more securely and effectively they move into single limb stance. Crowther et al. [30] also reported decreased movement at the hip with reduced extension during stance. Overall, the majority of abnormal changes present in the PAD patients seem to be at the hip and the ankle joints. These results are consistent with findings of Celis et al. [17] who also found an increase in the range of motion at the ankle during stance. However, Celis et al. [17] did not find significant differences at the hip. Chen et al. [2] found decreased hip flexion during early stance but did not report decreased range of motion at the hip during stance. Chen et al. [2] also reported increased ankle plantarflexion during stance in PAD patients as compared to controls. This agrees with the increased range of motion at the ankle during stance in PAD patients that was found in our study. The muscles surrounding the ankle, the gastrocnemius and soleus in particular, are the area of the leg most likely affected by ischemia since the blood must travel farther to reach the lower part of the leg. The abnormal biomechanical parameters at the ankle, including the decreased force production during late stance and the increased range of motion during stance, are affected by changes that occur further up in the kinetic chain at the hip joint. The increase in the range of motion of the ankle during stance was accompanied by a decrease in range of motion of the hip during stance as it was found in our study. This is logical when considering the entire kinetic chain. When motion at one joint is decreased, another joint in the kinetic chain may have to increase movement to maintain forward progression. Thus, it is possible that the ankle range of motion is increased before treatment as the hip movement is decreased as a compensation mechanism. The joint angle values found in this study are in general agreement with those reported in previous studies involving elderly populations [34,36].

Joint moment analysis showed no differences at the ankle before or after treatment. At the hip, weakness is observed in the hip flexors as evidenced by the decreased hip flexor moment (HFMM) in PAD patients. The weakness in the hip flexors would also account for the decreased hip flexion at heel contact and overall smaller hip range of motion, while decreased strength indicates that control of the ankle range of motion was reduced such that increased plantarflexion occurred at heel contact and increased dorsiflexion occurred prior to toe-off. Hip range of motion was decreased significantly in PAD patients which contributed to decreases in maximum hip flexor and extensor moments (Figure 2) that were noted before and after pharmacological treatment. Chen et al. [2] also found decreased peak hip extensor moment during stance in PAD patients compared to controls. This is likely due to decreased hip range of motion as was indicated in the joint angle results. The decreased hip moment values may also indicate an inability of PAD patients to generate sufficient muscular contractions at the hip during walking. Furthermore, the maximum knee extension moment is related with the development of the muscular contraction required to extend the knee and move the entire body over the straight leg during single support. Though this value was unchanged statistically, PAD patients may exhibit a decreased ability to generate this moment as indicated by the decreased knee extensor moment which was approaching significance.

The PAD patients in the current study also had a slower walking speed than healthy controls which is in agreement with other studies of PAD patients [4,7]. This altered gait speed suggests a baseline alteration in gait function in patients with PAD. It should be noted that despite pharmacological treatment in our current PAD cohort, no walking speed changes were observed.

The lack of changes in the biomechanical gait parameters with pharmacological treatment in the current study may be multi-factorial in nature. Reasons for lack of gait alterations may include treatment duration, medication compliance, and lack of drug efficacy. Nonetheless, gait adaptations may occur rapidly when changes are imposed on the musculoskeletal system. Several researchers found that supervised treadmill exercise over a 12-week period improved absolute claudication distances [37-39], improved peak oxygen uptake [38,39], and increased calf-muscle strength and calf-muscle endurance [39]. These results suggest that PAD patients may undergo significant changes with respect to muscle strength and walking ability within a 12 week period if treated with a supervised exercise program. Therefore, it is imperative that future studies examine the underlying mechanisms for improvement in gait parameters for both exercise and pharmacological treatment. If the mechanisms are found to be different based upon biomechanical analysis, combination treatment consisting of treadmill exercise and pharmacological treatment may be the most efficacious non-operative treatment for symptoms of PAD patients.

Several limitations for this study may explain the absence of significant changes found as a result of pharmacological treatment. It should be noted that during the biomechanical analysis of the kinematic and kinetic differences between PAD patients and controls and within PAD patients due to pharmacological treatment, walking speed was not controlled or analyzed as a covariate. Walking speed was not controlled during the data collections because it represents the natural self-selected walking speed of the populations. This decision is supported by research with elderly populations where it is common practice not to control for speed during biomechanical comparisons [31-34]. Next, pentoxyfilline was used as one of the treatment medications despite the fact that it has a variable effect when compared to placebo at improving walking distance in PAD patients [24]. This was unavoidable since it is one of the approved medications for treatment of PAD and is frequently prescribed for the treatment of intermittent claudication. Interestingly, pentoxyfilline has been shown to variably improve respiration capability in the mitochondria of skeletal muscle of PAD patients [14]. Thus it was not unreasonable to expect the drug to have an effect on the skeletal muscle of patients in this study and for those physiological changes to extend to gait alterations. Next, the medications were administered for a total of 12 weeks which is half the time of the Dawson et al. [24] study which found differences in walking distance due to pharmacological treatment. However, 12 weeks is the same amount of time that other studies involving PAD patients have used when pharmacological treatment and exercise therapy was employed and those studies did show significant improvements in walking distance [25,38]. The method of medication assignment was not ideal since the medications were not randomly assigned. It was not possible to randomly assign treatment groups since the patients recruited for this study had already been under the care of a physician for treatment of their PAD symptoms and the treating physicians had formulary restrictions regarding which medication would most benefit an individual patient. It should be noted that all patients in this study were combined into one group for analysis regardless of medication type. Because the focus of this study was not to evaluate the effects of one medication versus another, the groups were combined. However, based upon the literature and our current study, future studies should include sufficient patients to have two separate treatment groups for analysis. Importantly the results of our study are valid for non-strenuous walking. It is possible that the agents used here can elicit differences in the biomechanics of gait of PAD patients after being administered for a significant amount of time during strenuous walking such as when they exhibit ischemia. This question is presently being investigated in our laboratory. Furthermore, we should mention that the chronic ischemia reperfusion cycle which occurs in PAD results in damage to the mitochondria and overall neuromuscular damage of the lower extremity that is present even when patients are not experiencing claudication pain [11,12]. Finally, the control group was not re-tested after an equivalent passage of time experienced by the intervention group. The acquisition of a posttest observation for the control group would have permitted analysis with a 2 × 2 pre-test/post-test control group design and would have provided an ability to control for other confounding variables as well as the detection and evaluation of any interaction effect between groups and time/intervention.

## Conclusions

In conclusion, our study demonstrated that pharmacological treatment of PAD patients with intermittent claudication did not result in detectable changes in gait biomechanics during non-strenuous walking. Differences as compared to healthy controls remained before and after standard treatment with FDA approved drugs. Therefore, such treatment for PAD does not translate to biomechanical gait changes as hypothesized. The PAD gait variables showed a large number of significant differences when compared to controls both pre- and post-treatment. Future studies are needed to clarify whether cilostazol or pentoxifylline is more effective, from a biomechanical perspective, at improving gait during strenuous walking and if a longer period of administering the medication would have an effect on gait parameters. In addition, new pharmacotherapy options may be necessary in order to improve muscle function of the lower extremities and the claudication pain which limits the amounts of walking and other physical activity performed by PAD patients.

## Competing interests

We have read the submitted manuscript that includes our names as authors and vouch for its accuracy. We certify that we have participated sufficiently in the conception and design of this work and the analysis of the data (where applicable), as well as the writing of the manuscript, to take public responsibility for its content. We believe the manuscript represents honest and valid work. To the best of our knowledge, it contains no misrepresentations. We have reviewed the final version of the submitted manuscript and approve it for publication. We warrant that the manuscript is original and its essential substance, tables, or figures have not been previously published in part or in whole. The manuscript or one with substantially similar content under our authorship or the data within it has not been accepted for publication elsewhere and it is not presently under review by any other publisher. The manuscript will not be submitted for publication elsewhere until a decision has been made on its acceptability for publication in *Journal of NeuroEngineering and Rehabilitation*. This restriction does not apply to brief abstracts or press reports published in connection with scientific meetings.

## Authors' contributions

JH carried out gait analysis, performed data analysis/processing, performed statistical analysis, drafted the manuscript, and revision of the manuscript to the current version. IP participated in the design of the study, data interpretation, and approval of the manuscript version to be published. NS participated in the design of the study, data interpretation, manuscript revisions, and approval of the manuscript version to be published. JJ participated in data interpretation, manuscript revisions, and approval of the manuscript version to be published.
